# The genome sequence of the European golden eagle, *Aquila chrysaetos chrysaetos *Linnaeus 1758

**DOI:** 10.12688/wellcomeopenres.16631.1

**Published:** 2021-05-14

**Authors:** Dan Mead, Rob Ogden, Anna Meredith, Gabriela Peniche, Michelle Smith, Craig Corton, Karen Oliver, Jason Skelton, Emma Betteridge, Jale Doulcan, Nadine Holmes, Victoria Wright, Matt Loose, Michael A. Quail, Shane A. McCarthy, Kerstin Howe, William Chow, James Torrance, Joanna Collins, Richard Challis, Richard Durbin, Mark Blaxter

**Affiliations:** 1 Wellcome Sanger Institute, Hinxton, Cambridge, CB10 1SA, UK; 2 Owlstone Medical, Cambridge Science Park, Cambridge, CB4 0GJ, UK; 3 Royal (Dick) School of Veterinary Sciences and Roslin Institute, University of Edinburgh, Midlothian, EH25 9RG, UK; 4 Melbourne Veterinary School, University of Melbourne, Parkville, Victoria, 3010, Australia; 5 Achilles Therapeutics plc, London, W6 8PW, UK; 6 Deep Seq, University of Nottingham, Nottingham, NG7 2UH, UK; 7 Department of Genetics, University of Cambridge, Cambridge, CB2 3EH, UK

**Keywords:** Aquila chrysaetos, European golden eagle, genome sequence, chromosomal

## Abstract

We present a genome assembly from an individual female *Aquila chrysaetos chrysaetos* (the European golden eagle; Chordata; Aves; Accipitridae). The genome sequence is 1.23 gigabases in span. The majority of the assembly is scaffolded into 28 chromosomal pseudomolecules, including the W and Z sex chromosomes.

## Species taxonomy

Eukaryota; Metazoa; Chordata; Vertebrata; Aves; Accipitriformes; Accipitridae; Accipitrinae; Aquila; *Aquila chrysaetos* subspecies *chrysaetos* Linnaeus 1758 (NCBI:txid223781).

## Introduction

The golden eagle, *Aquila chrysaetos*, is an apex predator with a range that spans the Holarctic. It has been divided into six subspecies, with the nominate European subspecies, *A. chrysaetos chrysaetos* found across Europe, except for the Iberian peninsula, and extending eastwards in Russia as far as western Siberia. However, mitochondrial sequence and microsatellite analyses suggest that only two major clades exist within the species, a globally distributed northern clade and a distinct Mediterranean clade ( Nebel *et al.,* 2015; Nebel *et al.,* 2019; Sato *et al.,* 2017). Formerly widespread, *A. chrysaetos chrysaetos* is now confined to wilderness areas. Once found throughout Britain and Ireland, the golden eagle was extirpated from England and Wales by 1850 and in Ireland by 1912. The golden eagle was particularly badly impacted by bioaccumulating pesticides in the late 20th century ( Watson *et al.*, 2010). A single pair nested in the English Lake District from 1969–2004, but this has not led to a sustained recolonisation. There is ongoing monitoring of the remaining population in Scotland, where deliberate persecution is thought to be a major threat ( Fielding *et al.,* 2006).

## Genome sequence report

**Figure 1.**
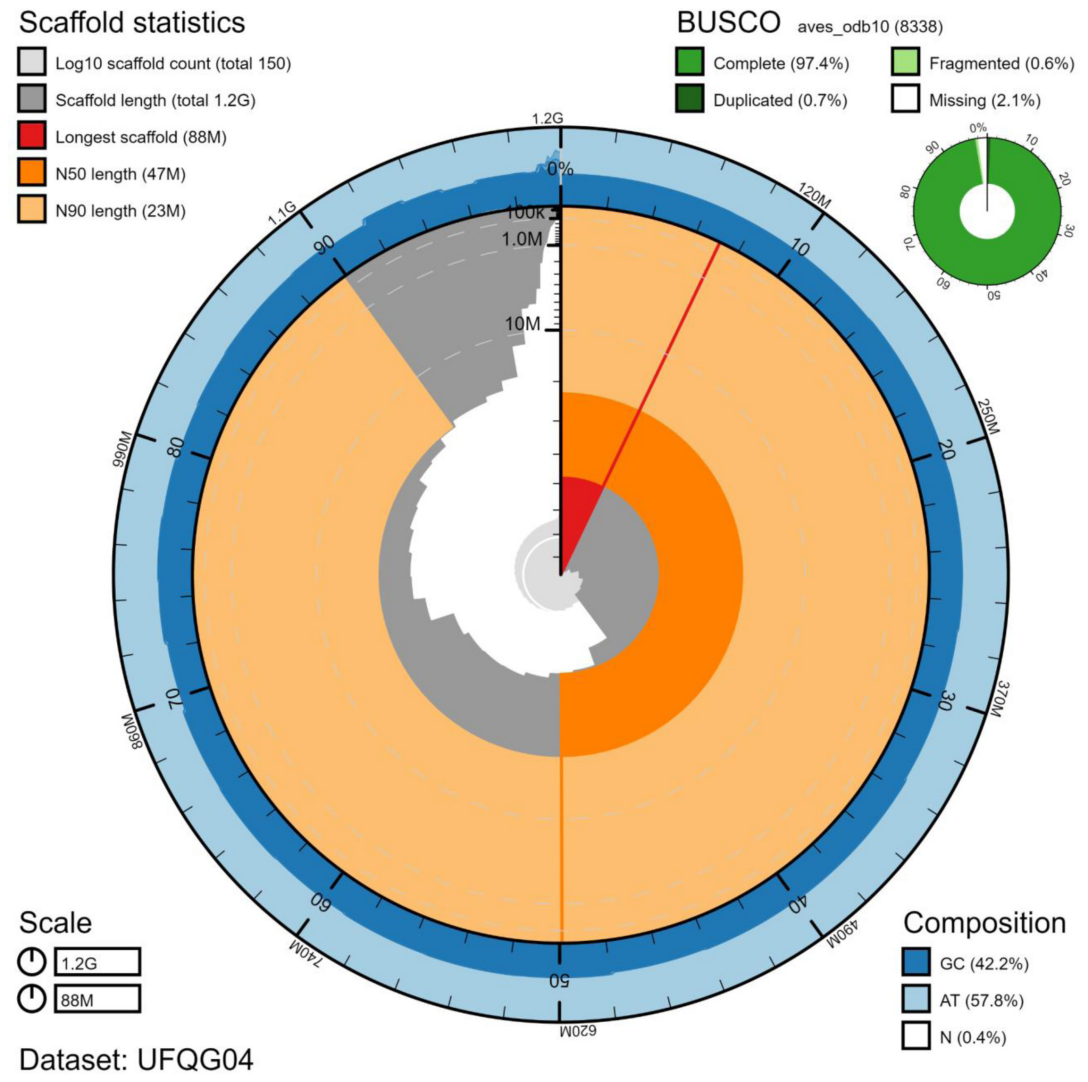
Genome assembly of *Aquila chrysaetos chrysaetos* bAquChr1.4. BlobToolKit Snailplot. The plot shows N50 metrics for bAquChr1.4 and BUSCO scores for the Aves set of orthologues. Interactive version available at https://blobtoolkit.genomehubs.org/view/Aquila%20chrysaetos%20chrysaetos/dataset/UFQG04/snail.

The genome was sequenced from a single female *A. chrysaetos chrytsaetos* collected by Gabriela Peniche under UK Home Office project licence PB8A1D5C7. A total of 46-fold coverage in Pacific Biosciences single-molecule long reads (N50 19 kb) and 47-fold coverage in 10X Genomics read clouds (from molecules with an estimated N50 of 68 kb) were generated. Primary assembly contigs were scaffolded with chromosome conformation HiC data. The HiC scaffolds were validated using BioNano long-range restriction maps (140-fold effective coverage in molecules of N50 310 kb). The final assembly has a total length of 1.23 Gb in 145 sequence scaffolds with a scaffold N50 of 46.9 Mb ( Table 1). The majority, 99.0%, of the assembly sequence was assigned to 28 chromosomal-level scaffolds representing 26 autosomes (numbered by sequence length), and the W and Z sex chromosomes ( Figure 1– Figure 4; Table 2). The assembly has a BUSCO ( Simão *et al.,* 2015) completeness of 97.4% using the aves_odb10 reference set. While not fully phased, the assembly deposited is of one haplotype. Contigs corresponding to the second haplotype have also been deposited. The *A. chrysaetos chrysaetos* assembly has equivalent span and scaffold-level contiguity to an assembly of *A. chrysaetos canadiensis* (NCBI:txid216574) produced by the Erez Lieberman Aiden lab as part of the DNAZoo project.

**Table 1.** Genome data for *Aquila chrysaetos chrysaetos* bAquChr1.4.

*Project accession data*	
Assembly identifier	bAquChr1.4
Species	*Aquila chrysaetos chrysaetos*
Specimen	GE037-17
NCBI taxonomy ID	223781
BioProject	PRJEB27699
Biosample ID	SAMEA994725
Isolate information	Female, heart muscle tissue
*Raw data accessions*	
PacificBiosciences SEQUEL I	ERR2980431, ERR2980432, ERR2980435, ERR2980436, ERR2980437, ERR2980438, ERR2980439, ERR2980440, ERR2980448, ERR2980449, ERR2980450, ERR2980451, ERR2990043, ERR2990044, ERR3013207, ERR3013208
10X Genomics Illumina	ERR3316065, ERR3316066, ERR3316067, ERR3316068
Hi-C Illumina	ERR3312497
BioNano	ERZ1392826
*Genome assembly*	
Assembly accession	GCA_900496995.4
*Accession of alternate* *haplotype*	GCA_902153765.2
Span (Mb)	1,233
Number of contigs	373
Contig N50 length (Mb)	22
Number of scaffolds	145
Scaffold N50 length (Mb)	47
Longest scaffold (Mb)	85
BUSCO * genome score	C:97.4%[S:96.7%,D:0.7%], F:0.6%,M:2.1%,n:8338

* BUSCO scores based on the aves_odb10 BUSCO set using v5.0.0. C= complete [S= single copy, D=duplicated], F=fragmented, M=missing, n=number of orthologues in comparison. A full set of BUSCO scores is available at https://blobtoolkit.genomehubs.org/view/Aquila%20chrysaetos%20chrysaetos/dataset/UFQG04/busco.

**Figure 2.**
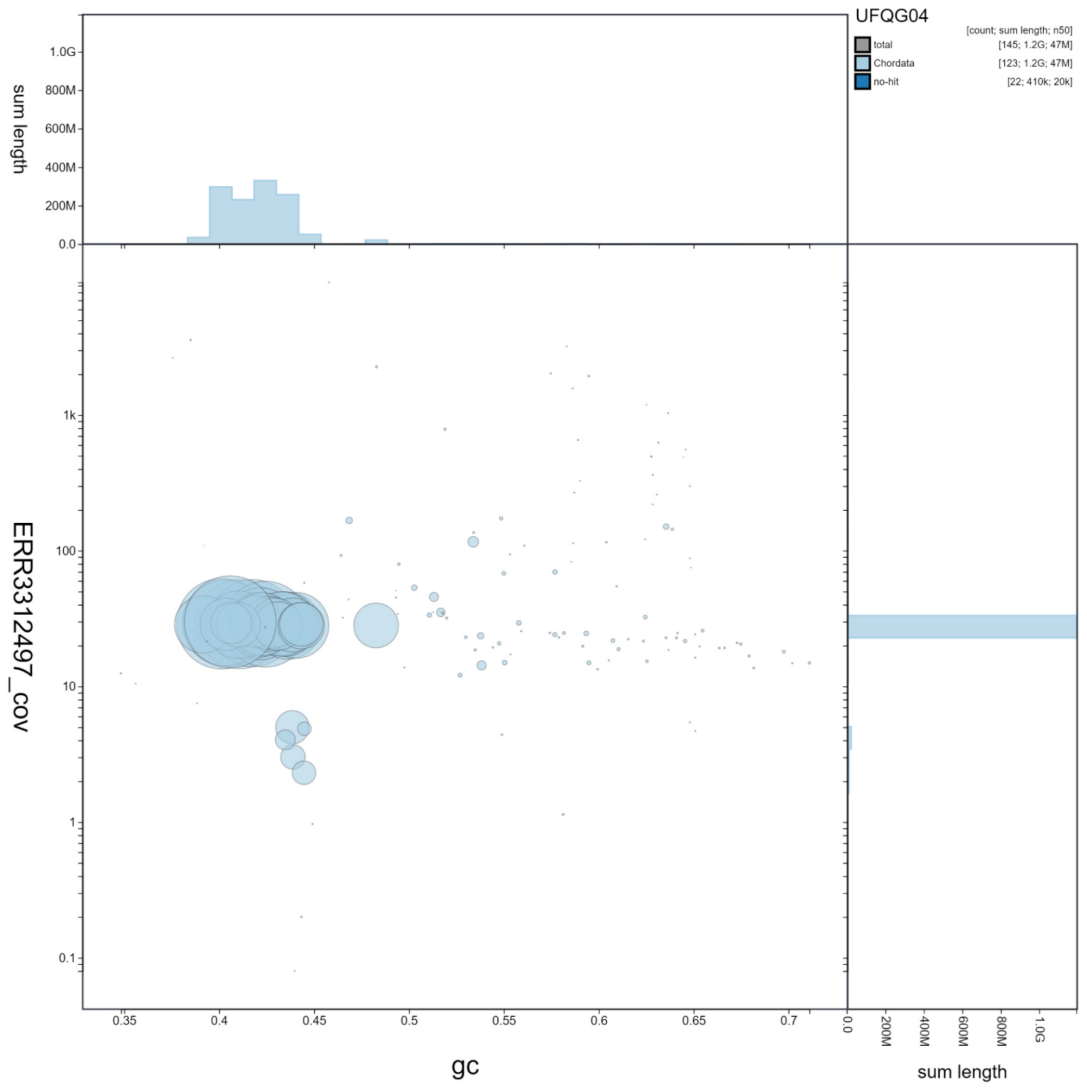
Genome assembly of *Aquila chrysaetos chrysaetos* bAquChr1.4. BlobToolKit GC-coverage plot. Interactive version available at https://blobtoolkit.genomehubs.org/view/Aquila%20chrysaetos%20chrysaetos/dataset/UFQG04/blob?plotShape=circle.

**Figure 3.**
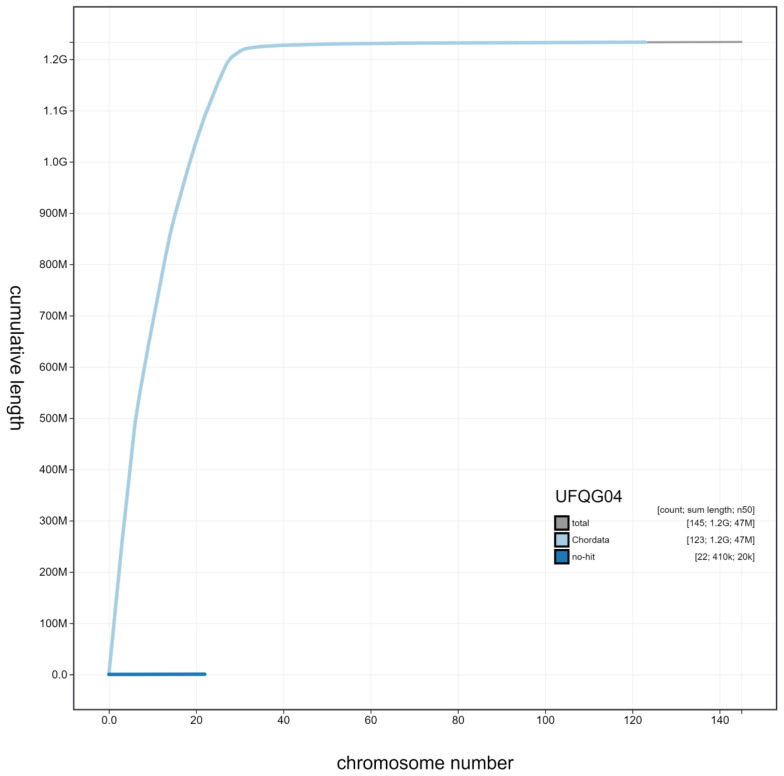
Genome assembly of *Aquila chrysaetos chrysaetos* bAquChr1.4. BlobToolKit Cumulative sequence plot. Interactive version available at https://blobtoolkit.genomehubs.org/view/Aquila%20chrysaetos%20chrysaetos/dataset/UFQG04/cumulative.

**Figure 4.**
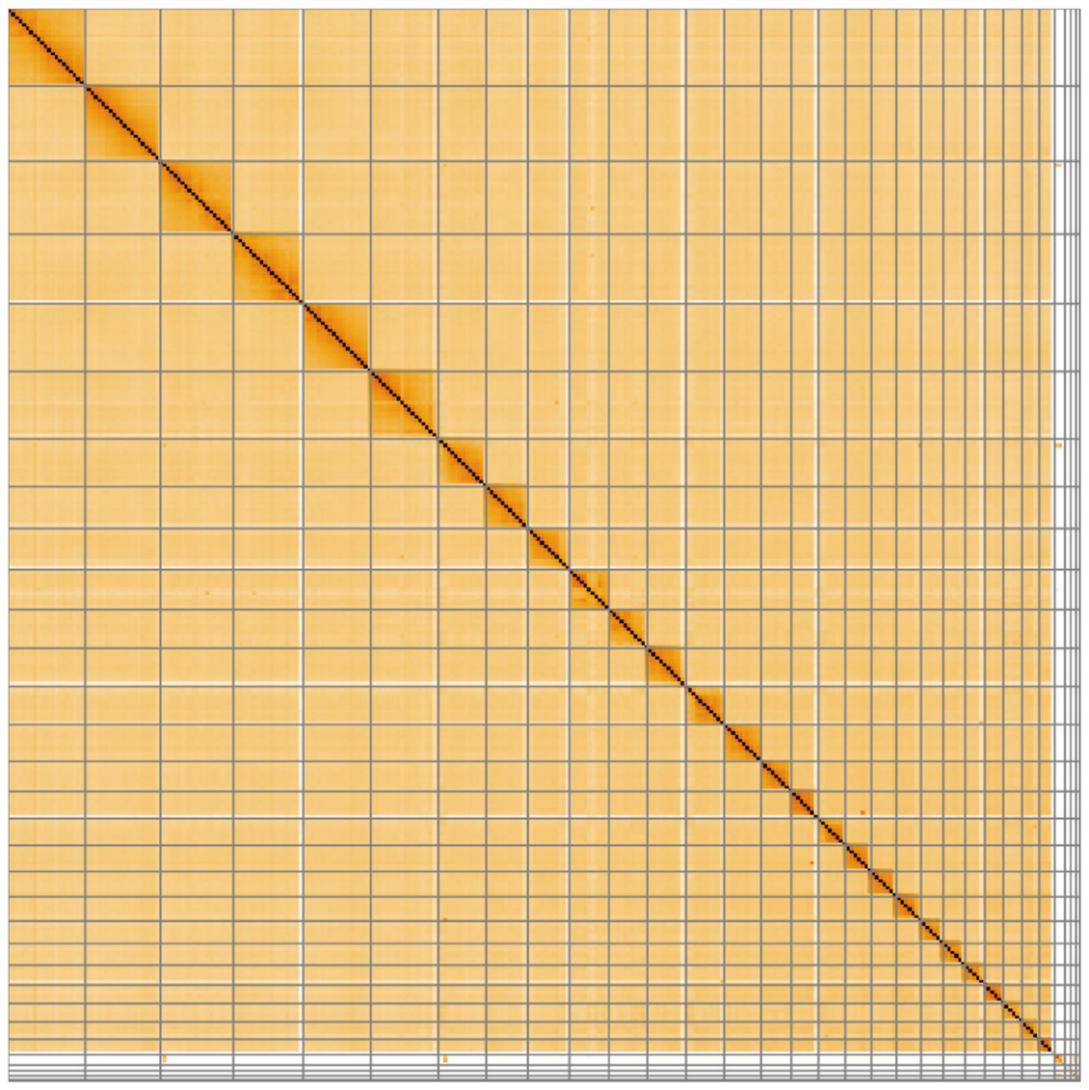
Genome assembly of *Aquila chrysaetos chrysaetos* bAquChr1.4. Hi-C contact map. Hi-C contact map of the bAquChr1.4 assembly, visualized in HiGlass.

**Table 2.** Chromosomal pseudomolecules in the genome assembly of *Aquila chrysaetos chrysaetos* bAquChr1.4.

*ENA accession*	*Chromosome*	*Size (Mb)*	*GC%*
LR606181.1	1	85.46	40.2
LR606182.1	2	83.00	41.1
LR606183.1	3	79.38	41.8
LR606184.1	4	77.27	40.4
LR606185.1	5	76.62	42.5
LR606186.1	6	54.40	42.2
LR606187.1	7	47.78	41
LR606188.1	8	46.94	43.6
LR606189.1	9	45.24	44.1
LR606190.1	10	43.95	43.5
LR606191.1	11	43.76	42.2
LR606192.1	12	43.48	43.3
LR606193.1	13	41.79	42.3
LR606194.1	14	34.34	39.2
LR606195.1	15	30.98	42.7
LR606196.1	16	30.61	43.9
LR606197.1	17	29.70	42.6
LR606198.1	18	28.56	40.4
LR606199.1	19	27.98	41.9
LR606200.1	20	25.31	43.1
LR606201.1	21	24.76	43.1
LR606202.1	22	22.51	44.3
LR606203.1	23	21.01	41.1
LR606204.1	24	20.99	48.3
LR606205.1	25	19.84	44.3
LR606206.1	26	17.72	40.7
HG999777.1	W	11.73	44.1
LR606180.1	Z	88.22	40.7
-	unplaced	30.39	48.4

As the Hi-C data was sourced from a male bird, it was not possible to fully construct the W chromosome for the female sample bAquChr1. Scaffolds identified as belonging to W have therefore been submitted as unordered fragments. The largest of these fragments has been designated as the W Chromosome and all other W scaffolds labelled as W_unloc.

## Methods

The golden eagle specimen was collected, following death by natural causes, from an area 15 km from the Highland village of Fort Augustus, Scotland, under UK Home Office project licence no. PB8A1D5C7. The heart was dissected out during autopsy. The specimen is preserved frozen at the University of Edinburgh.

DNA was extracted from heart tissue following the BioNano protocol. Pacific Biosciences CLR long read and 10X Genomics read cloud sequencing libraries were constructed according to manufacturers’ instructions. Sequencing was performed by the Scientific Operations core at the Wellcome Sanger Institute on Pacific Biosciences SEQUEL I and Illumina HiSeq X instruments. Hi-C data were generated using the Dovetail HiC library preparation kit at WSI.

BioNano data were generated in DeepSeq, University of Nottingham. High Molecular Weight genomic DNA (HMW gDNA) was extracted from an agarose plug (bAquChr (Golden Eagle); Plug 2) that had been prepared and shipped according to the Bionano Agarose Plug Shipping Instructions. The Bionano Prep Animal Tissue DNA Isolation Soft Tissue Protocol (Document Number: 30077; Document Revision: C) was used to complete HMW gDNA extraction. DNA quantitation, using the Qubit Fluorometer and the Qubit dsDNA BR kit (ThermoFisher; Q32853), gave a mean concentration of 117 ng/ul (CV = 0.118). Labelling was performed with an input of 750 ng of HMW gDNA, using the DLS DNA Labeling Kit (Bionano: 80003) and the Bionano Prep Direct Label and Stain (DLS) Protocol (Document Number: 30206; Document Revision: D). The labelled sample was quantified by Qubit Fluorometer and the Qubit dsDNA HS Assay Kit (ThermoFisher: Q32854). The average concentration of the labelled sample was 4.37 ng/ul (CV = 0.042). The labelling reaction was run over one flowcell of a Bionano Saphyr Chip (Bionano: 20319) on the Bionano Saphyr (Bionano; 60239) running software versions - Bionano Access: 1.2.2; Bionano Tools: 7921; Bionano Solve: Solve3.2.2_08222018; RefAligner: 7782.7865rel; HybridScaffold/SVMerge/VariantAnnotation: 08222018. For analysis, the molecule file was used to generate a *de novo* assembly using the default Bionano Access settings. This assembly was used to generate a hybrid scaffold from the reference ufqg01.fasta. The hybrid scaffold was constructed using default settings; conflict resolution was set to ‘Resolve Conflicts’ for both the Bionano assembly and sequence assembly.

Assembly was carried out using Falcon-unzip (falcon-kit 1.1.1) ( Chin *et al.,* 2016), haplotypic duplication was identified and removed with purge_dups ( Guan *et al.,* 2020) and a first round of scaffolding carried out with 10X Genomics read clouds using scaff10x. Hybrid scaffolding was performed using the BioNano DLE-1 data and Bionano Solve v3.3. The Hi-C scaffolded assembly was polished with arrow using the PacBio data, then polished with the 10X Genomics Illumina data by aligning to the assembly with longranger align, calling variants with freebayes ( Garrison & Marth, 2012) and applying homozygous non-reference edits using bcftools consensus. Two rounds of the Illumina polishing were applied. The assembly was checked for contamination and manually corrected using the gEVAL system ( Chow *et al.,* 2016; Howe *et al.*, 2021). This reduced the sequence length by 2.2% and the scaffold count by 44.7% whilst increasing the scaffold N50 by 4.4%. The genome was analysed within the BlobToolKit environment ( Challis *et al.,* 2020). Software versions are given in Table 3.

**Table 3.** Software tools used.

Software tool	Version	Source
Falcon-unzip	falcon-kit 1.2.2	( Chin *et al.,* 2016)
purge_dups	1.0.0	( Guan *et al.,* 2020)
SALSA2	2.2	( Ghurye *et al.,* 2019)
scaff10x	4.2	https://github.com/wtsi-hpag/Scaff10X
arrow	GenomicConsensus 2.3.3	https://github.com/PacificBiosciences/GenomicConsensus
longranger align	2.2.2	https://support.10xgenomics.com/genome-exome/software/ pipelines/latest/advanced/other-pipelines
freebayes	v1.1.0-3-g961e5f3	( Garrison & Marth, 2012)
bcftools consensus	1.9	http://samtools.github.io/bcftools/bcftools.html
HiGlass	1.11.6	( Kerpedjiev *et al.,* 2018)
PretextView	0.0.4	https://github.com/wtsi-hpag/PretextView
gEVAL	N/A	( Chow *et al.,* 2016)
BlobToolKit	2.5	( Challis *et al.,* 2020)

##  Data availability

### Underlying data

European Nucleotide Archive: Aquila chrysaetos chrysaetos (European golden eagle) genome assembly, Accession number PRJEB33202 https://www.ebi.ac.uk/ena/browser/view/PRJEB33202.

The genome sequence is released openly for reuse. The *A. chrysaetos chrysaetos* genome sequencing initiative is part of the Wellcome Sanger Institute’s “ 25 genomes for 25 years” project. It is also part of the Vertebrate Genome Project (VGP) ordinal references programme and the Darwin Tree of Life (DToL) project. The specimen has been preserved at Edinburgh University and will be deposited in CryoArks. All raw data and the assembly have been deposited in the ENA. The genome will be annotated and presented through the Ensembl pipeline at the European Bioinformatics Institute. Raw data and assembly accession identifiers are reported in Table 1.
