# Supplementary material for: The genome sequence of the European golden eagle, Aquila chrysaetos chrysaetos Linnaeus 1758
Source: Wellcome Open Res. 2021 May 14;6:112. [Version 1] doi: 10.12688/wellcomeopenres.16631.1 (PMC8499043; doi:10.12688/wellcomeopenres.16631.1)
Supplement: Peer review history

# Peer review: Reviewer response for version 1

**DOI:** 10.21956/wellcomeopenres.18335.r46304

**Published:** 2021-11-15

**Authors:** Xiang-jiang Zhan, Zhongru Gu

**Affiliations:** 1 Key Lab of Animal Ecology and Conservation Biology, Institute of Zoology, Chinese Academy of Sciences, Beijing, China

This data note presents the chromosomal genome assembly of the European golden eagle, *Aquila chrysaetos chrysaetos. *Although it was not the first golden eagle genome assembly reported *, *the authors denovo sequenced a new individual using hybrid techniques including PacBio, 10X Genomics, Hi-C and BioNano. It will facilitate the comparative and population genomics analysis for golden eagle in future. In general, the report was clearly written and the authors did a good job for this top predator. I have only two comments:

1. There are several inconsistencies in the description of software. The version of Falcon-unzip is inconsistent between the method part (falcon-kit 1.1.1) and Table 3 (1.2.2). The SALSA2 listed in Table 3 is not mentioned in the method. The Bionano Solve (v3.3) is mentioned in the method, but not in Table 3.
2. The authors could increase the font size in figure 2 and 3.

Are sufficient details of methods and materials provided to allow replication by others?

Partly

Is the rationale for creating the dataset(s) clearly described?

Yes

Are the datasets clearly presented in a useable and accessible format?

Yes

Are the protocols appropriate and is the work technically sound?

Yes

Reviewer Expertise:

Conservation genetics of raptorial birds

We confirm that we have read this submission and believe that we have an appropriate level of expertise to confirm that it is of an acceptable scientific standard.

# Peer review: Reviewer response for version 1

**DOI:** 10.21956/wellcomeopenres.18335.r46301

**Published:** 2021-10-07

**Authors:** Carles Lalueza-Fox

**Affiliations:** 1 Institute of Evolutionary Biology (CSIC-Universitat Pompeu Fabra), University of Barcelona, Barcelona, Spain

This is a comprehensible and well-written report on the generation of a golden eagle female specimen. Golden eagles have ecological importance for being top predators among birds, but also as religious and political icons along history in many different regions and countries. Their genome can provide the basis for further research on their specific adaptations. The details of the sequencing effort and the quality of the assembly and annotation are sound; overall, I think this would be a valuable resource for the scientific community.

 I only have a minor comment; maybe the authors should cite a previous work (Doyle *et al.* 2014 ^1^) where a male from another subspecies ( *A*. *c*. *canadiensis*) from Sierra Nevada (USA) was sequenced in 2,552 scaffolds. Obviously, due to technological improvements, the current annotation is much better, but future researchers could likely use the previous one to explore inter subspecies diversity.

 Also, as a minor comment, Linnaeus 1758 should be between brackets.

Are sufficient details of methods and materials provided to allow replication by others?

Yes

Is the rationale for creating the dataset(s) clearly described?

Yes

Are the datasets clearly presented in a useable and accessible format?

Yes

Are the protocols appropriate and is the work technically sound?

Yes

Reviewer Expertise:

Evolutionary Genomics, Paleogenomics

I confirm that I have read this submission and believe that I have an appropriate level of expertise to confirm that it is of an acceptable scientific standard.

## References

[1] : The genome sequence of a widespread apex predator, the golden eagle (Aquila chrysaetos). *PLoS One* .2014;9(4) : 10.1371/journal.pone.0095599 e95599 10.1371/journal.pone.0095599 24759626PMC3997482

# Peer review: Reviewer response for version 1

**DOI:** 10.21956/wellcomeopenres.18335.r43937

**Published:** 2021-06-10

**Authors:** Bengt Hansson

**Affiliations:** 1 Department of Biology, Lund University, Lund, Sweden

I think this is a well-written Data Note, which presents an important resource for further genetic analyses of the golden eagle and related species. I have only two comments.

1. First, the focus on UK and Ireland in the Introduction seems a bit arbitrary for a general readership. Similar stories about population declines can be applied to several countries. Perhaps, simply, a well-placed “For example” (or similar) would be enough?
2. Secondly, the HiC individual is not included in Table 1, which then provides incorrect information.

Are sufficient details of methods and materials provided to allow replication by others?

Yes

Is the rationale for creating the dataset(s) clearly described?

Yes

Are the datasets clearly presented in a useable and accessible format?

Yes

Are the protocols appropriate and is the work technically sound?

Yes

Reviewer Expertise:

Population genomics and genetics of birds

I confirm that I have read this submission and believe that I have an appropriate level of expertise to confirm that it is of an acceptable scientific standard.
